# 18F-FDG-PET/MR in Alveolar Echinococcosis: Multiparametric Imaging in a Real-World Setting

**DOI:** 10.3390/pathogens11030348

**Published:** 2022-03-12

**Authors:** Nina Eberhardt, Lynn Peters, Silke Kapp-Schwoerer, Meinrad Beer, Ambros J. Beer, Beate Grüner, Wolfgang M. Thaiss

**Affiliations:** 1 Department of Nuclear Medicine, Ulm University Hospital, Albert-Einstein-Allee 23, 89081 Ulm, Germany; nina.eberhardt@uniklinik-ulm.de (N.E.); ambros.beer@uniklinik-ulm.de (A.J.B.); 2 Department of Internal Medicine III, Division of Infectious Diseases, Ulm University Hospital, Albert-Einstein-Allee 23, 89081 Ulm, Germany; lynn.peters@uniklinik-ulm.de (L.P.); silke.kapp-schwoerer@uniklinik-ulm.de (S.K.-S.); beate.gruener@uniklinik-ulm.de (B.G.); 3 Department of Diagnostic and Interventional Radiology, Ulm University Hospital, Albert-Einstein-Allee 23, 89081 Ulm, Germany; meinrad.beer@uniklinik-ulm.de; 4 Core Facility PET/MR, Medical Faculty, University Ulm, Albert-Einstein-Allee 23, 89081 Ulm, Germany

**Keywords:** alveolar echinococcosis, FDG-PET, PET/MR, MRI, liver imaging

## Abstract

Recent improvements in alveolar echinococcosis (AE) therapy can provide long-term disease control, and even allow structured treatment interruption in selected cases. Imaging has a pivotal role in monitoring disease activity, with 18-fluoro-deoxyglucose positron emission and computed tomography (18F-FDG-PET/CT) in particular having proven beneficial for assessing disease activity. Repetitive regular examinations to monitor therapy response, however, can lead to substantial radiation burden. Therefore, by combining metabolic information and excellent tissue contrast in magnetic resonance imaging (MRI), PET/MR appears ideally suited for this task. Here, we retrospectively analyzed 51 AE patients that underwent 18F-FDG-PET/MR. Patients had a ‘confirmed/probable’ diagnosis in 22/29 cases according to the WHO classification. FDG uptake, diffusion restriction, and MRI morphology were evaluated. We found significant differences in FDG uptake between responders to benzimidazole therapy and progressive manifestations (SUVavg 2.7 ± 1.3 vs. 5.4 ± 2.2, *p* < 0.001) as well as between Kodama Types 1 and 3 (F = 9.9, *p* < 0.003). No significant differences were detected for ADC values or MRI morphology concerning response and no correlations were present between FDG uptake and ADC values. The mean radiation dose was 5.9–6.5 mSv. We conclude that the combination of metabolic information and MRI morphology at a low radiation dose proposes PET/MR as a suitable imaging modality for AE assessment. Longitudinal studies are needed to define the role of this imaging modality.

## 1. Introduction

As a rare parasitic disease, alveolar echinococcosis (AE) affects countries in the Northern Hemisphere, especially the western regions of China, Western Europe, and Turkey, as well as eastern Russia, North America, and Japan [1,2,3]. Prognosis is poor when left untreated, with a mortality close to 100% after 10 years [4,5]. With the liver being the main target organ, the parasitic manifestation can lead to failure in liver function and spread beyond the liver.

With the introduction of benzimidazoles (BZM) in the late 1970s, survival rates gradually improved, especially when supplemented with surgery as a possible long-term therapeutic option. However, as BZM have a parasitostatic effect, they require continued and permanent medication in most cases. Eventually, successful treatment strategies can lead to long-term remission and the BZM therapy might be discontinued (structured treatment interruption, STI) [6,7]. However, there is no single laboratory or imaging marker that can assess the vitality of the hepatic foci. The patients need to be monitored for an extended period of time, even after surgical resection of all previously visible liver lesions. Therefore, beside clinical parameters, imaging approaches have become essential not only for the assessment of distribution and surgical or conventional therapy planning, but especially for the monitoring of therapy response in AE.

Laboratory assessment is essential both for the initial diagnosis and for defining response to therapy. Initially, a genus-specific ELISA serves to detect antibodies to *Echinococcus* spp., which are also utilized to assess the therapeutic success together with imaging markers [8,9,10]. The results of further subsequent ELISAs detecting *E. multilocularis*-specific antibodies to Em2+ or EM18 correlate with active disease and can therefore serve to monitor disease activity. Adequate therapeutic response is reflected in seroconversion, especially after complete surgical resection [11].

**Figure 1:**
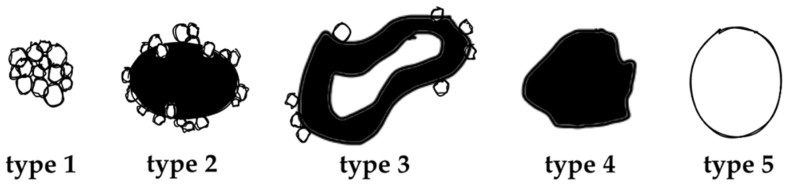
Alveolar Echinococcosis lesion type according to Kodama’s MRI classification, adapted from [19].

The value of (contrast-enhanced) ultrasound (US), computed tomography (CT), and magnetic resonance imaging (MRI) has been demonstrated extensively for disease distribution and description of AE lesions [12,13,14,15,16,17,18]. Especially the excellent tissue contrast in MRI enables detailed lesion characterization and led to the development of a classification system based on morphological features, first described by Kodama et al. [19]. Based on characteristic and distinct lesions appearances on MRI, they defined five different types of hepatic AE manifestations. Type 1 comprises grouped small cystic lesions; Type 2 consists of a central solid compartment that is surrounded by smaller cystic components: Type 3 has, additionally to Type 2, a larger central cystic lesion; Type 4 consists solely of a larger solid component; and Type 5 is defined as a large pure cystic lesion (Figure 1). The clinical relevance of Kodama et al.’s lesion classification is based on the biological representation of the course of the disease. While an initial stage is mostly represented by very small cystoid lesions, the disease progression is mostly represented with Type 1 and Type 2 lesions. Advancements during the course of (untreated) disease are oftentimes manifested in Type 3 lesions, with a continuous transition to regressive stages in Types 4 and 5 [20].

While CT and MRI can provide valuable information on disease distribution, morphological changes do not always accurately represent viable residual disease or can change only minimally even under response to treatment, thus hampering further treatment stratification. Metabolic activity visualized in 18-fluoro-deoxyglucose positron emission tomography (18F-FDG-PET) has been shown to provide additional information on disease activity and thereby aiding clinical decision making on the continuation of therapy or surgery planning [21,22,23]. This led to the recommendation for 18F-FDG-PET/CT by the WHO informal working group for echinococcosis to assess disease activity when available [8].

The combination of PET and CT, however, involves substantial radiation for patients that undergo yearly or biyearly controls in a non-resectable state of disease before STI can be tried, especially if contrast-enhanced CT is used. Low-dose CT in combination with PET imaging can reduce the radiation dose; however, this comes at the cost of reduced lesion delineation when compared to contrast-enhanced CT. Thus, in clinical routine we introduced PET/MR in feasible cases. Here we report the first data of and experiences in the clinical work-up of AE patients using FDG PET/MR. Therefore, we investigated in a well-characterized cohort of patients the value of hybrid PET/MR in addition to clinical parameters.

## 2. Results

### 2.1. Patients’ Characteristics

Fifty-one consecutive AE patients that underwent 18F-FDG-PET/MR were analyzed. Mean age at diagnosis was 46.5 ± 15.3 years and mean age at 18F-FDG-PET/MR examination was 51.3 ± 15.8 years. Fourteen patients (27.5%) were between ages 18–40, 20 (39.2%) were in the 41–60 age group, and 17 (33.3%) were over 60 years old. Six patients had no prior 18F-FDG-PET/CT examination and two of those were staged at the time point of diagnosis. Seventeen patients had one prior 18F-FDG-PET/CT and 28 ≥ 2 prior 18F-FDG-PET/CT examinations.

AE diagnosis based on histological or PCR-criteria according to World Health Organization—Informal Working Group on Echinococcosis recommendations was ‘confirmed’ in 22 cases and ‘probable’ in 29 cases. Response to therapy was present in 44 patients (33 stable under BZM, and 11 stable without therapy) and four patients showed signs of progressive disease as determined by combined clinical, laboratory, and imaging findings. All four had a measurable increase in lesion size and showed enhanced FDG-uptake. Serologic analysis revealed high activity in all four patients. The two patients with PET/MR staging at the time of diagnosis did not receive any previous medication. Three patients had undergone previous partial liver resection (2, 37, and 272 months prior to PET/MR). Laboratory values at the time of investigation are summarized in Table 1, and patients’ PNM stages are summarized in Table 2.

### 2.2. Kodama’s MRI Classification

**Figure 2:**
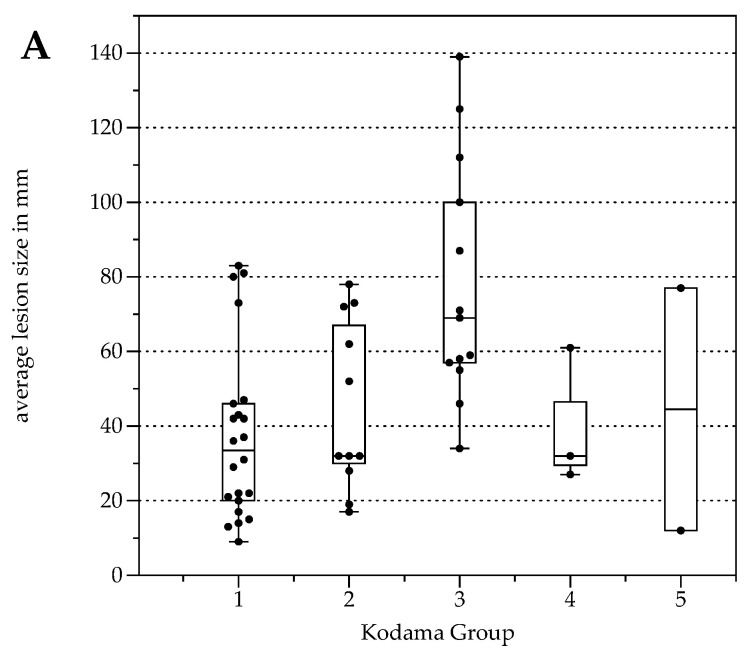

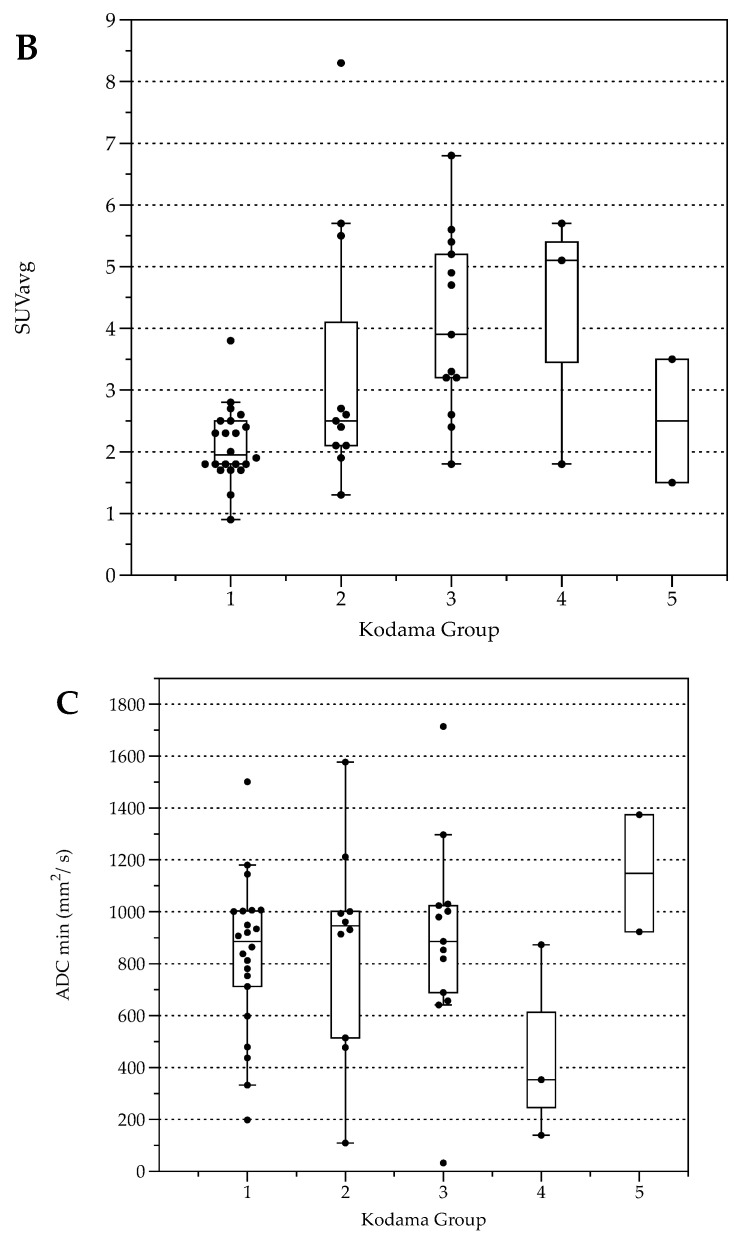
Box plots with distribution of average lesion size (**A**), average Standardized Uptake Values SUVavg (**B**), and minimal Apparent Diffusion Coefficient (ADCmin) (**C**) grouped by morphologic MRI appearance according to Kodama et al. [19].

**Figure 3:**
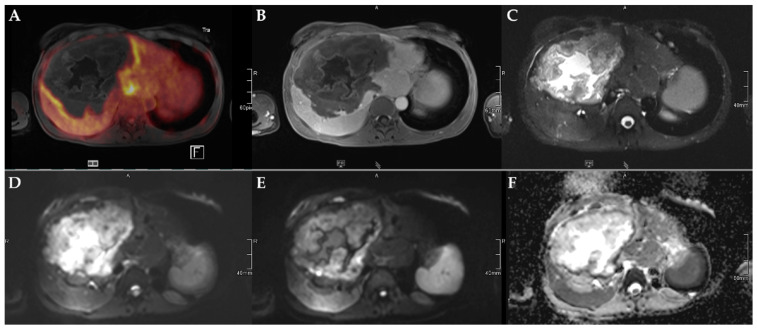
45-year-old female patient with AE and involvement of liver segments IVa–VII and Kodama Type 3 lesion. Increased FDG uptake is observed at the lesion rim (**A**) with increased uptake of the contrast agent (**B**). High signal intensity is present in the central cystic parts in T2w imaging with fat suppression (**C**). Diffusion imaging demonstrates a high signal at a low b-value (b50) in the central parts of the lesion (**D**), a low signal at a high b-value (b800) (**E**), and high ADC values (**F**), indicative of no diffusion restriction.

**Figure 4:**
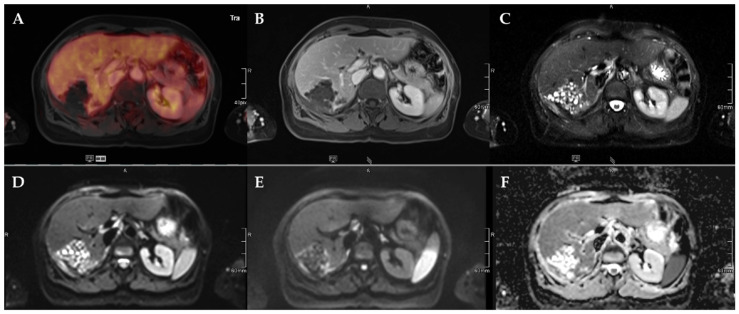
72-year-old female patient with AE and a Kodama Type 1 lesion in liver segments VII and VIII. No increased FDG uptake is observed (**A**) and no relevant contrast enhancement (**B**). High signal intensity in T2w imaging with fat suppression illustrates the small cystic character of the Type 1 lesion (**C**). Diffusion imaging demonstrates a high signal at a low b-value (b50) in the central parts of the lesion (**D**), a low signal at a high b-value (b800) (**E**), and high ADC values (**F**), indicative of no diffusion restriction.

The number of lesions was 22 (Type 1), 11 (Type 2), 13 (Type 3), 3 (Type 4), and 2 (Type 5, Table 3). Lesion size was significantly different between Kodama Types 1 vs. 3 and 2 vs. 3 (F = 5.4, *p* < 0.001 and <0.05, respectively, Figure 2A). SUVavg values were 2.1 ± 0.6 for Type 1, 3.4 ± 2.2 for Type 2, 4.1 ± 1.5 for Type 3, 4.2 ± 2.1 for Type 4, and 2.5 ± 1.4 for Type 5. Significant differences between Groups 1 and 3 were detected for SUVavg (F = 9.9, *p* < 0.003) and SUVmax (2.6 ± 0.8 and 5.0 ± 1.7, F = 9.3, *p* < 0.04). Patient examples are given in Figure 3 and Figure 4.

### 2.3. Lesion Characterization by FDG Uptake and Diffusion Restriction

**Figure 5:**
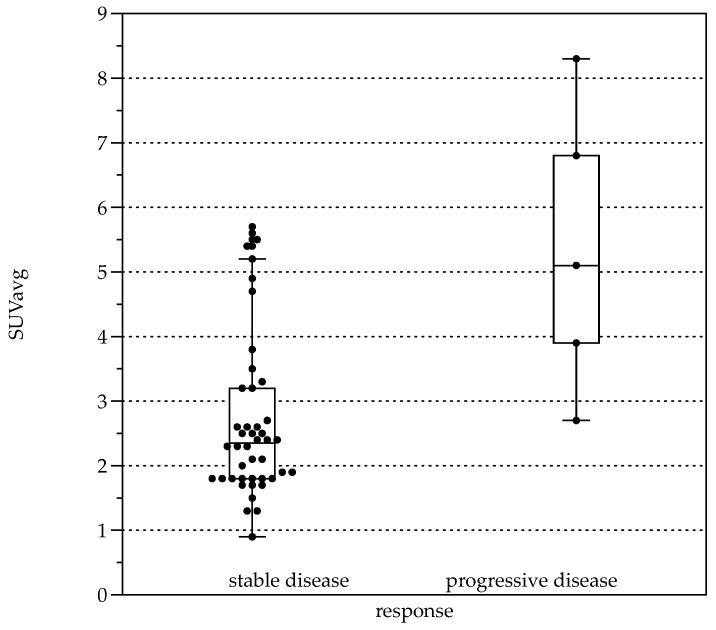
Boxplots for average Standardized Uptake Value (SUVavg) in patients with response to therapy (left, stable disease with and without BZM (*n* = 44, SUVavg 2.7 ± 1.28) and patients with progressive disease (right, *n* = 5, SUVavg 5.36 ± 2.24, *p* < 0.001).

Average FDG uptake for all the examinations, expressed in Standardized Uptake Values (SUV), was 3.0 ± 1.6 (SUVavg). Significant differences were present comparing patients with response to therapy (stable disease with and without BZM, *n* = 44, SUVavg 2.7 ± 1.3, SUVmax values 3.3 ± 1.6) and patients with progressive disease (*n* = 5, SUVavg values 5.4 ± 2.2, *p* < 0.001 and SUVmax 6.6 ± 2.8, *p* < 0.001, Figure 5). When stratified by Kodama’s groups, significant differences were present between Groups 1 and 3 (F = 10.5, *p* < 0.01, Figure 2B).

For all lesions, ADCavg was 1.2 ± 0.3 × 10^−3^ mm^2^/s, and ADCmin 0.8 ± 0.4 × 10^−3^ mm^2^/s. ADCavg and ADCmin were not significantly different between patients with response to therapy and progressive disease or between Kodama’s MRI lesion type. No correlation was detected between SUVavg and SUVmax, nor between ADCavg and ADCmin (r = 0.03, *p* = 0.04 and r = 0.2, *p* = 0.2, respectively). This was also true for the corrected SUV values when with the lesion-to-liver background ratios. However, lesions with solid parts tended to have lower ADCmin values, especially Group 4 (Figure 2C), although no group differences were present between the Kodama groups.

SUVavg was moderately correlated with the Em2 antigen status (r = 0.45, *p* = 0.001) and IgG levels (r = 0.62, *p* < 0.0001).

### 2.4. FDG-PET Radiation Dose

With a mean injected dose between 310 to 340 MBq for 18F-FDG, the mean radiation dose per patient was between 5.9 and 6.5 mSv when a conversion factor of 0.019 mSv/MBq is used.

## 3. Discussion

In this study we retrospectively evaluated PET/MR examinations in a clinically well characterized patient cohort with AE, which to our knowledge is the largest series so far. We found significant differences between patients with progressive disease and stable disease as well as between Kodama Types 1 and 3 regarding FDG uptake but not for ADC values, which suggests that complementary information is provided by PET and DWI. Thus, due to excellent multiparametric molecular profiling of the AE lesion extent and activity and reduced radiation exposure compared to PET/CT, PET/MR mostly replaced PET/CT in our clinical routine workup of AE.

The Kodama classification described lesion types based on morphological features and therefore defined recognizable classes that made AE lesions comparable between different studies. Cystic features therein represent metacestodal vesicles and liquefaction necrosis, whereas solid parts consist of necrosis, calcifications, and granulomatous tissue [19,20]. The lesion type at presentation seems to be dependent on several factors, with time of disease progression before clinical presentation and speed of lesion growth being among the factors that explain interpatient and regional differences. In our cohort, lesion Types 4 and 5 were a minority, and lesion Types 1 and 2/3 accounted for 43% and 47%, respectively. While Types 2 and 3 were also dominant in most studies, including the initial publication by Kodama et al. [19,20,24], the overrepresentation of Type 1 lesions in our study might be due to the overall relatively small lesion size with a mean in Type 1 lesions of 37.1 mm, which is in line with larger investigations (e.g., 35.7 mm in [20]) and the relatively early time of diagnosis.

Lesions with solitary components tended to have diffusion restriction, which is reflected in Kodama Types 2 and 4. This is in line with previous reports; for example, Becce et al. [24] identified significant correlations between the presence of solid lesion components and ADCmin. Pure cystic lesions, i.e., Kodama Type 5, tend to have the highest ADC values also in our cohort, which can be expected due to their non-restricted diffusivity within the large cystic lesion. We did not find significant group differences in this regard when correcting for multiple testing (Figure 2C); however, this might mainly be due to the small number of patients in the Types 4 and 5 groups.

MRI provides excellent soft-tissue contrast and characterization of lesion type, relationship to larger vessels, the biliary ducts, and possible obstruction, as well as identification of extrahepatic extension. However, early therapy response assessment based on changes in lesion size is not feasible with MRI alone, which is better represented in FDG uptake. Thus, the WHO suggests the use of 18F-FDG-PET/CT for assessment of disease activity and several studies provided evidence for the value of this examinations in AE [8]. Reuter et al. [25] defined 18F-FDG-PET/CT as a sensitive and specific method adding value for diagnosing suspected AE, which was confirmed in consecutive studies [26]. Later the same group distinguished a further indication for 18F-FDG-PET/CT in AE with the demonstration of ancillary information in the decision for STI [21,27]. Recently, Amann et al. also showed that therapy discontinuation after normalized 18F-FDG-PET/CT in conjunction with anit-EmII/3–10 levels led to intermediate term recurrence-free states in 11 patients [21]. These findings established today’s role of 18F-FDG-PET/CT during therapy monitoring and BZM termination and have been confirmed recently over an extended period of time [22]. This approach can be transferred to PET/MR as the acquired FDG information is comparable, as has been demonstrated multiple times in the past [28]. An early comparative study between modalities was assessed by Reuter et al. [12], demonstrating the advantages of AE screening with ultrasound and the value of CT for covering the true extent of the disease and characteristic calcifications. The conjunct nature of 18F-FDG-PET/CT and MRI for AE assessment has been shown by Azizi et al. [29], investigating patients that underwent both MRI and 18F-FDG-PET/CT. They found correlations between FDG uptake and the presence of microcystic lesions on MRI, namely, Types 1, 2, and 3 lesions. Our results show moderate variations from their findings as we also found comparably high FDG uptake in Type 4 lesions. This might be due to the solid nature of those lesions; however, both studies are biased due to the low number of lesions for type 4 (*n* = 3 in [29], *n* = 3 in [19], and *n* = 4 in our study) and a comparably smaller lesion size.

With regard to PET/MR, Lötsch et al. [30] reported an initial experience with four PET/MR examinations in AE. They conclude that this modality is suited for AE examinations with significantly reduced radiation burden. The positive development with favorable disease control over the last decades [2] has increased the demand for long-term monitoring of disease activity in non-resectable disease and thus the demand for 18F-FDG-PET studies. With a typical control interval between 6, 12, and 24 months [21,22,26], the cumulative radiation dose from CT and PET can be substantial for a non-oncologic disease. With PET/MR, the radiation dose can be reduced to the FDG portion while still providing the beneficial soft tissue contrast in MRI and the availability of dynamic contrast-enhanced images, thereby supplementing additional MRI examinations between 18F-FDG-PET/CT acquisitions. With a mean radiation dose of between 5.9 and 6.8 mSv in our cohort, the average reduction compared to 18F-FDG-PET/CT is around 8 mSv lower compared to the standard contrast-enhanced PET/CT effective dose protocols and might be even higher when additional examination phases are added; e.g., arterial liver examinations [31]. Especially with regard to the relatively young age at initial diagnosis (55% below the age of 50 years), the cumulative reduction in repetitive examinations can be substantial. Whether there is a need for an MRI contrast agent in AE follow-up exams is a separate issue beyond the scope of this article, but potential effects for repeated Gadolinium expositions need to be considered.

The limitations of the current study include the retrospective nature of the investigation, and the different time from diagnosis to imaging and therapy. Limiting also is the low number of patients with progressive disease at the time of examination. We only assessed larger lesions and measured the visually highest FDG-avid regions; however, this was a common strategy in similar examinations [29,32]. No long-term follow-up for individual patients is provided due to the relatively recent start of regular 18F-FDG-PET/MR examinations.

The described benefits of MRI contrast and the possibility to assess disease activity by means of glucose metabolism in parallel without the radiation from CT has prompted us to utilize 18F-FDG-PET/MR for staging examinations. We are aware that, currently, this approach is not applicable across a large scale and only accessible at selected centers, and that this is an initial study. However, outside oncological applications AE might be an ideal indication for 18F-PET/MR, especially for long-term follow-up examinations. Whether PET/MR can furthermore provide additional information on clinical decision making beyond the established combination of FDG uptake and morphological information derived from MRI by utilizing multiparametric features, needs to be addressed in future studies.

## 4. Material and Methods

### 4.1. Study Population

In total, 51 consecutive patients (34 female) that underwent FDG-PET/MR at our institution between 08/19 and 07/21 were included in this retrospective analysis. Clinical data on patients with AE were collected from the German National Echinococcus database at Ulm University Hospital. Patient characterization was carried out as previously described [33]: At their first visit, patients had either findings suggestive of AE or were already under treatment for manifest AE. The diagnosis was ascertained by US, CT, or MRI; immunodiagnosis with standard commercial tests, enzyme-linked immunosorbent assay; confirmatory serology with crude antigens, Em10, and antigen B, and histopathology with haematoxylin-eosin staining, PAS, and immunohistological staining. Since 2000, a staging examination with FDG-PET additionally has been applied. PET imaging is routinely performed for primary staging at time of diagnosis and biyearly under medication. In patients that previously underwent surgery, PET imaging is indicated two years after surgery and before discontinuation of BZM. The specific indication for PET imaging was disease monitoring under BZM or clinically suspected disease progression (*n* = 49). All patients that were planned for PET/CT during follow up were offered a PET/MR examination starting 08/19 as an alternative (lower radiation dose and superior tissue contrast in MRI compared to CT) if no contraindications were present, such as metallic implants, claustrophobia, or known allergy to the MRI contrast agent. Initial staging for suspected echinococcus infection was done with PET/MR in two cases as requested by a patient aged 33 and in a patient with abdominal CT four weeks before planned PET assessment.

Repeated PET/MR examinations were not included in this analysis. Only patients diagnosed with ‘confirmed’ or ‘probable’ AE according to the WHO case definition were included [8]: ‘probable’ indicates a typical clinical presentation, epidemiological history, imaging findings, and serology positive for AE, while the ‘confirmed’ label additionally requires histopathology compatible findings with AE and/or E. multilocularis-nucleic acid sequences derived from a clinical specimen. Progressive disease was defined as increase in lesion size, new intra- or extrahepatic manifestations, or AE-associated complications such as new or enhancing cholestasis. Response to therapy was defined as lesion stability or decrease in size and the absence of clinical complications as well as new intra- or extrahepatic manifestations.

### 4.2. Imaging Study

Patients were scanned with a 3 T PET/MR scanner (Biograph mMR, Siemens Healthcare, Erlangen, Ergmany) after injection of 18F-FDG 60 min prior to the examination. Patients had to fast at least for six hours prior to injection. The injected doses were adapted by body weight (between 310 to 340 MBq). In total, 10 mg Furosemid were injected for faster renal clearing of FDG. Gadobutrol was used as the contrast agent (Gadovist, Bayer AG, Leverkusen, Germany) and adapted by body weight (1 mL/10 kg body weight).

The imaging protocol consisted of a T1w DIXON in axial orientation for attenuation correction, T2w HASTE STIR axial, T1w CAIPI axial post Gadolinium injection for imaging of mid thorax to thighs if patients had previous examinations with no evidence of thoracic or brain lesions. In the case of initial staging, head to thighs were included. Additionally, a focus region in the upper abdomen included diffusion weighted imaging (DWI) with two b-values (b50, b800), which were used to calculate the apparent diffusion (ADC) maps, dynamic contrast enhancement with a Flash 3d axial, and T2w HASTE in the axial and coronal orientation.

### 4.3. Image Analysis

Lesion size and location were documented using the PNM classification, with P0–4 describing the extent of the parasitic mass in the liver, N0–1 the spread to neighboring organs, and M0–1 the presence of distant metastases [1,4]. The PNM classification is deduced from the TNM classification used for tumor staging. Lesion size measurements were performed with an IMPAX EE (R20 XVIII SU1, Agfa HealthCare, Mortsel, Belgium). Regions of interest were drawn at the border of the lesions with the highest FDG uptake; the corresponding ROIs were used for ADC-measurements in syngo.via (VB40B, Siemens Healthineers, Erlangen, Germany). In cases of multiple lesions, the lesion with the highest FDG uptake was chosen for the analysis. Lesions were analyzed by two experienced nuclear medicine physicians/radiologists. For Kodama’s MRI classification, a consensus reading was done on T2w images in case of discrepancies between readers.

### 4.4. Serological Testing

IgG screening ELISA (Institut Virion/Serion GmbH, Würzburg, Germany) were carried out on a fully automated DS2 ELISA platform (Dynex Technologies, Chantilly, USA), as described in [10]. Em2+ ELISA (Bordier Affinity Products SA, Crissier, Switzerland) was interpreted qualitatively (positive/negative) according to the manufacturer’s instructions. Total IgE was measured using an electrochemiluminescence immunoassay on a Cobas e801 platform (Roche Diagnostics Deutschland GmbH, Mannheim, Germany).

### 4.5. Statistical Analysis

Statistical analysis was performed with R (R version 4.0.4, R Core Team (2013). R: A language and environment for statistical computing. R Foundation for Statistical Computing, Vienna, Austria. ISBN 3-900051-07-0, URL http://www.R-project.org/, accessed on 15 January 2022). The ‘pastecs’ and ‘psych’ packages were used for descriptive statistics (frequency, minimum, and maximum, mean, standard deviation (SD). For correlations, we used the Pearson correlation coefficient. Group differences were determined using a one-way analysis of variance (ANOVA) with post-hoc Bonferroni correction. *p*-values < 0.05 were interpreted as statistically significant. Values are given as the mean ± SD if not stated otherwise. DataGraph 4.7.1 (Visual Data Tools) was used for Box Plots.

## Figures and Tables

**Table 1:** Characteristics of the study population.

	No.
Age at diagnosis (mean, range)	46.6, 17–73
IgE levels (mean, range)	331.5, 7.7–5728.0
IgG levels (mean, range)	50.9, 0–252.0
ELISA Em2+ (positive/negative)	39/12

**Table 2:** PNM staging of AE.

	No.
Liver involvement, N0 M0	
P1	2
P2	5
P3	11
P4	12
PX, N1 and M0	15
PX, NX and M1	6

**Table 3:** AE lesion type according to Kodama’s MRI classification.

Lesion Type	No. of Lesions	No. of Lesions with SUVavg > 2.5	No. of Lesions with Diffusion Restriction < 0.8 × 10^−3^ mm^2^/s	Mean Lesion Size in Millimeter, Range
Type 1	22 (43%)	6	6	37, 9–83
Type 2	11 (22%)	6	3	45, 19–27
Type 3	13 (25%)	11	1	139, 34–139
Type 4	3 (6%)	2	1	40, 27–61
Type 5	2 (4%)	1	0	44, 12–77

AE: alveolar echinococcosis; MRI: magnetic resonance imaging; SUVavg: Standardized Uptake Value average, lesion size in millimeter.

## Data Availability

Data available on request due to privacy and ethical restrictions.
